# Anthrax in one health in Southern and Southeastern Europe - the effect of climate change?

**DOI:** 10.1007/s11259-023-10238-3

**Published:** 2023-10-21

**Authors:** Viorica Railean, Jarosław Sobolewski, Jędrzej M. Jaśkowski

**Affiliations:** 1 Department of Infectious, Invasive Diseases and Veterinary Administration, Institute of Veterinary Medicine, Nicolaus Copernicus University in Torun, Gagarina 7, Toruń, 87-100 Poland; 2 Centre for Modern Interdisciplinary Technologies, Nicolaus Copernicus University, Wilenska 4, Torun, 87-100 Poland; 3 Department of Public Health and Animal Welfare, Institute of Veterinary Medicine, Nicolaus Copernicus University, Gagarina 7, Toruń, 87-100 Poland; 4 Department of Diagnostics and Clinical Sciences, Institute of Veterinary Medicine, Nicolaus Copernicus University, Gagarina 7, Toruń, 87-100 Poland

**Keywords:** *Bacillus anthracis*, Virulent factor, Biological weapon, Epidemiology, Zoonosis

## Abstract

*Anthrax* is a serious infection caused by *Bacillus anthracis*. The anthracis spores are highly resistant and can persist in the environment for several decades. Therefore, anthrax is considered a global health threat affecting wildlife, livestock, and the general public. The resistance mechanism is influenced not only by the environment or the ecological niche but also by virulence factors. In the last 10 years the Southern and Southeastern Europe have been confronted with this threat. Recently, there have been 8 human anthrax cases reported in Croatia (2022), and 4 cases in Romania (2023). Moreover, this incident and the COVID situation could be a starting point to encourage researchers to raise the alarm. On the other hand, climate change is causing glaciers to melt and land to thaw, and many wetlands and swampy areas are being drained. It should not be forgotten that epidemiological and epizootic threats significantly affect the country’s economic development. The Covid-19 epidemic best illustrates these threats.

## Anthrax as an etiological agent

Anthrax was discovered by Robert Koch in 1876 and became the first bacterium to be experimentally shown as a pathogen (Blevins and Bronze 2010). *Bacillus anthracis* produce highly resistant spores that can persist in the environment for several decades. Therefore, it is a considerable global health threat affecting wildlife, livestock, and the general public (Manzulli et al. 2017). Phylogenetically, *Bacillus anthracis* belongs to the *Bacillus cereus* group, from the *Bacillus* genus and the *Bacillaceae* family, which comprises seven closely related species: *B. cereus sensu stricto* (referred to here as B. cereus), *B. anthracis, B. thuringiensis, B. mycoides, B. pseudomycoides, and B. cytotoxicus* (Ehling-Schulz et al. 2019). These species are similar in phenotypic and genetic properties; greater than 99% sequence similarity in the primary structures of 16 S rRNA exists among these species. However, they differ significantly in their extrachromosomal genetic elements, e.g. the production of virulent factors. In contrast to other species in the group, *B. anthr*acis strains exhibit low genetic diversity, although isolates can be differentiated by the characterization of variable number tandem repeats or amplified nucleotide polymorphisms in the genome (Weekes and Kotra 2007).

The microbiology of *Bacillus anthracis* exists in two forms: the vegetative form and the spore. The vegetative form is facultatively anaerobic, has square-ended rods, approximately 1 by 5–8 μm, with the propensity to form chains and Gram-positive bacillus, which, under adverse growth conditions, forms a single subterminal endospore (Koehler 2009). The formation of endospores is influenced by temperature, pH, moisture, and the presence of oxygen and carbon dioxide. In vitro studies have demonstrated that *B. anthracis* spores will germinate at 8–45 ˚C, pH 5–9, relative humidity > 95%, and with adequate nutrition (e.g. in the presence of L-alanine). Therefore, in adverse environmental conditions, e.g. released into the soil from a dead or dying animal, the vegetative bacilli die, but endospores survive, and in such way become resistant to the environmental conditions (e.g. temperature, pH, chemicals, or irradiation) and, consequently, create disinfection difficulties (Schmid and Kaufmann 2002).

Moreover, the resistance mechanism will be influenced not only by the environment or ecological niche but also by the bacterial species and their immune system, and, in turn, the resistance factor will be directly associated with virulence. Thus, in some cases, increased resistance is accompanied by increased virulence. However, in other cases, the increased resistance reduces the virulence of the microorganisms (Cepas and Soto 2020).

**Fig. 1:**
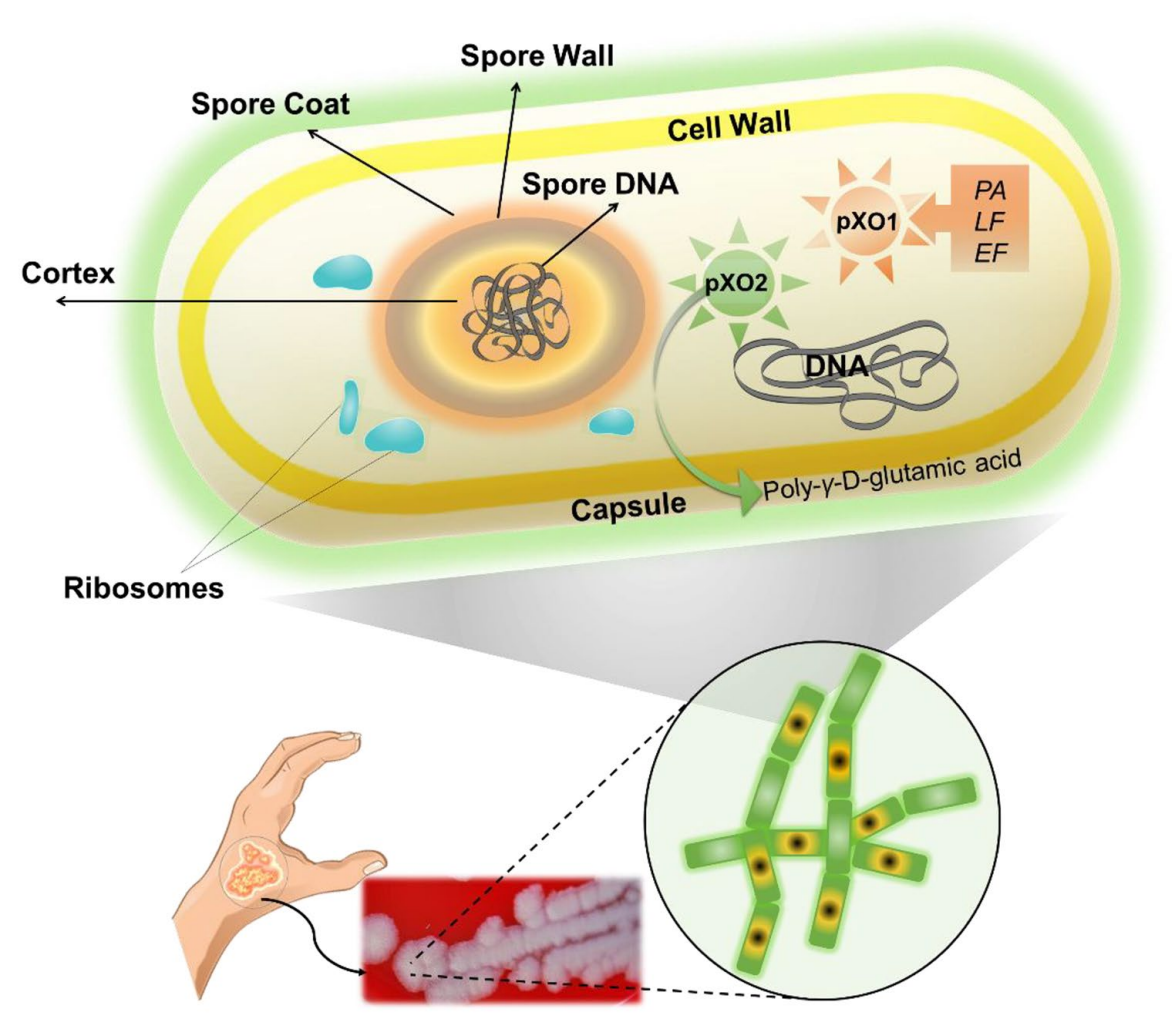
Bacillus anthracis - virulence factors; protective antigen (PA), lethal factor (LF) and edema factor (EF)

*Bacillus anthracis* possesses three primary virulence factors: an extracellular poly-D-glutamate (D-PGA) capsule, the lethal factor (LF), and edema factor (EF), all of which are coded on one of two plasmids, the pX01 and pX02 plasmids (Fig. 1).

Consistent with their large size, the virulence plasmids are maintained at a low copy number, with three genes required for pXO1 plasmid partitioning. Whole-genome sequencing revealed a 3:2:1 molecular ratio of pXO1: pXO2:chromosome in *B. anthracis* cells. (Moayeri et al. 2015). *B. anthracis* transconjugants carrying pXO12 or pXO14 can serve as donors of pXO1 and pXO2. Transfer of the virulence plasmids appears to occur via conduction. Important to specify is the fact that some transfer genes carry mutation or frameshifts (Koehler 2009).

Moreover, the diverse global dissemination of this species has also been described, indicating that many lineages may be uniquely suited to the geographic regions. According to the genotyping approach (e.g. Canonical Single Nucleotide Polymorphism (CanSNPs) method, Multiple-locus variable-number tandem repeat analysis (MLVA), Allelic expression etc.), the isolates are subdivided into three previously recognized major lineages (A, B, and C). The lineages A represent the strains reported worldwide, while the B and C lineages belong to the restricted regions. For instance, the B.Br.CNEVA sublanguage is ecologically established in the middle of Europe, spreading from the southwest of France to the northeast of Poland along the Italian Alps, Switzerland, Germany, Croatia and Slovakia. Trans-Eurasian group (TEA) is one of the most common groups of the *B. anthracis* in the world (Europe, Russia, Kazakhstan, the Caucasus). In recent decades, most cases have been found in Spain, Bulgaria, Greece, Romania, Afghanistan and Turkey (Derzelle and Thierry 2013). Moreover, recently, in 2023, 4 cases have been reported in Romania.

Recently, the *B. anthracis* genotype distribution has been reported in the Ukrainian territory and neighboring regions. The investigated strains were found to fall into the A lineage of the global phylogeny and clustered together with some strains that were isolated from Russia and Bulgaria. In contrast, some strains dominated in Eastern Europe, Rumania, Norway, Slovenia and Kazakhstan. In turn, the *B. anthracis* population found in Poland was more diverse (Derzelle and Thierry 2013). Therefore, the discrepancies between isolates result from the geographic origin and are most likely the result of homoplasy caused by the higher mutation rate.

The properties of *Bacillus anthracis* make this bacterium dangerous to human and animal health and life, not only in areas where it is endemic. In addition, the ease of multiplication of this biological pathogen in laboratory conditions and the storage and transport of spore forms make this microorganism even a model element of biological weapons and a zoonosis risk factor.

## Anthrax in Southern and Southeastern Europe

Anthrax is a disease known to humanity for centuries. Today, few medical and veterinary practitioners have had the opportunity to come into direct contact with the disease (Beyer and Turnbull 2009). It is worth noting that the latest cases of anthrax were reported in Europe. It evolves so fast that during one year − 107 dead cows and 8 human infection cases in Croatia (July 2022) and 3 cases in Romania (July 2023) have been reported. In July (2022), the Croatian Ministry of Agriculture announced the appearance of the disease among cattle in pastures located in the “Lonjsko Polje” Nature Park (Pole Lonja). All of the infected patients had come into contact with infected animals and were treated at the hospital. Each of the patients showed symptoms of a mild, cutaneous form of the disease. The infections occurred as a result of coming into contact with sick cattle, according to the portal Vijesti. Anthrax rarely spreads onto humans and is rarely transmitted from person to person. About 95% of all cases of anthrax in humans result from skin contact with infected animals. Untreated cases can be fatal.

According to the criteria of the Birds Directive of the European Union, the park is an important bird habitat (Important Bird Area - IBA). This oblong area is located southeast of Zagreb, in a floodplain situated on the Sava River, known for its unique environment. The Pole Lonja is the largest protected wetland in both Croatia and the entire Danube basin; covers an area of 505.6 square kilometers, stretching along the Sava River. The average annual amount of precipitation is 872 mm, while the average annual temperature is 9.5^o^C (Goel 2015). Anthrax spores can lie dormant in the ground until they are swallowed by animals or activated when the soil is disturbed by a heavy rain, flooding, or drought. Epidemics can kill large numbers of animals in a short time. Infected livestock is often found dead without the disease being detected (Goel 2015).

On the other train of thought, a fatal case of anthrax in a cow was reported in Croatia in 2007 in Bobovać near Sunja. It is worth emphasizing that death was caused not by the lack of the antibiotic therapy, but by a vaccine with insufficient spore content (Habrun et al. 2011).

As to the reported situtation in Romania, in addition to the 3 cases mentioned above, during the epidemiological investigation, another 20 people were identified who possessed meat or meat products from the respective animal. All these people have been medically monitored and so far do not show symptoms of illness (https://flutrackers.com/forum/).

**Fig. 2:**
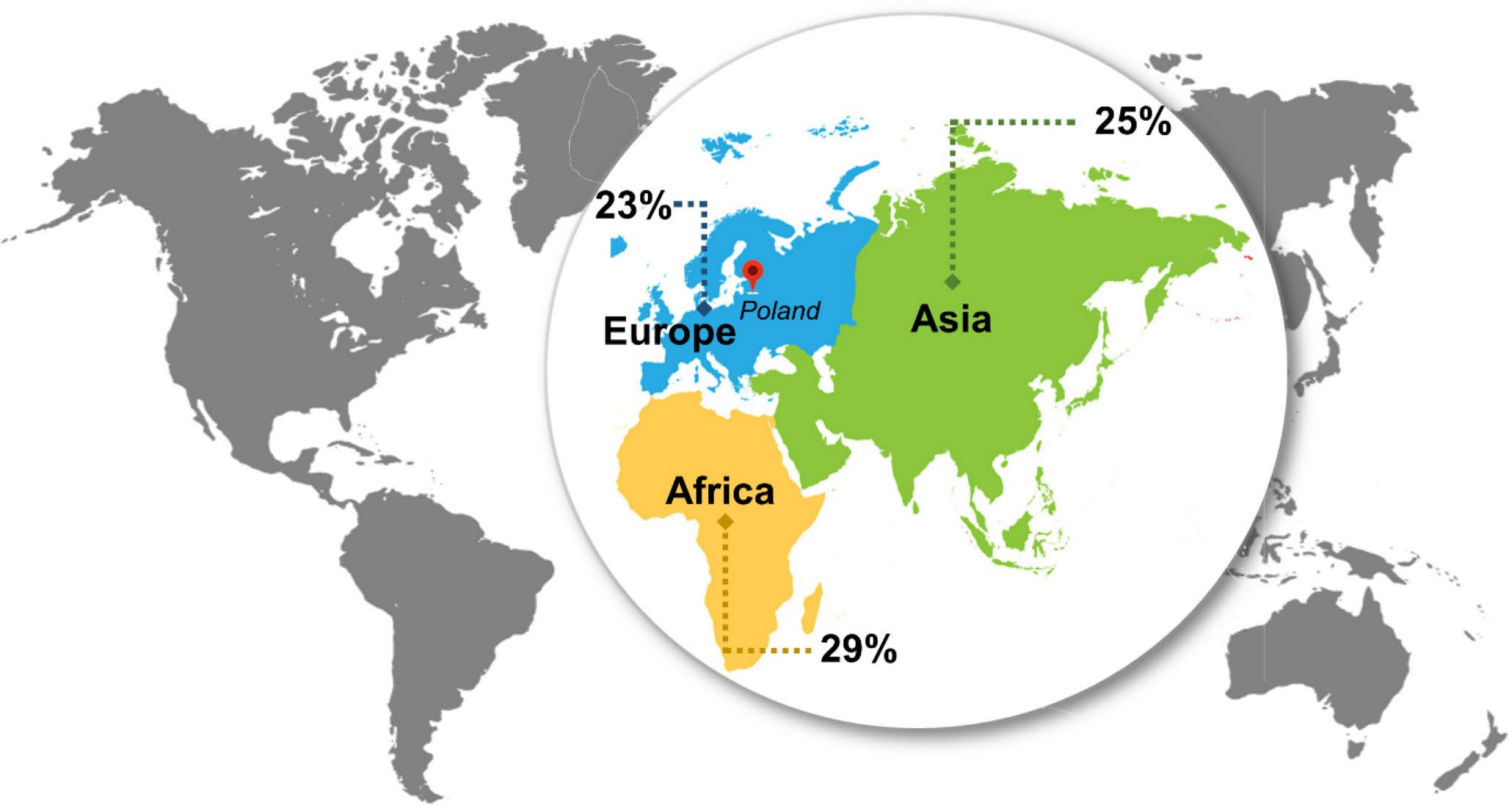
Map presenting the prevalence of anthrax for the period from 1992 to 2020

Nowadays, such incidents encourage researchers and veterinarians to be more focused on anthrax. Even from 2021, Bylaiah Sushma and his research group, have been sounding the alarm by studying the world prevalence of anthrax from 1992 to 2020. According to their report (Fig. 2), the highest prevalence of anthrax was estimated in Africa (29%), followed by Asia (25%) and Europe (23%) (Sushma et al. 2021).

Even if Europe is at the end of the list, the number of incidents does not differ much from Asia. This fact encourages people to pay more attention to anthrax as it is considered to be dangerous for both humans and livestock. Moreover, it has been demonstrated that Southern and Southeastern Europe in the last 10 years have been confronted with such a threat. Acording to the Kozytska 2023, the highest numbers of registered anthrax cases were: 28 cases in Albania, 24 in Russia, 23 in Italy, 20 in Romania and France, 18 in Moldova, followed by Greece and Hungary with 16 registred cases, Montenegro – 15 cases, Bulgaria – 14 cases, Ukraine – 13 cases and Croatia – 12 cases.

Anthrax cases were reported in almost all European countries and the highest numbers of reported cases were seen in 2016, 2018, and 2021 (Kozytska et al. 2023).

For instance, in Slovenia, the last recorded case of anthrax was on August 21, 2015. One year later, a mandatory, nationwide program funded by the government was implemented. Suspicion of anthrax is based on clinical symptoms or post-mortem examination.

The veterinarian reporting the suspicion of anthrax takes blood samples from live animals or sends the carcasses for laboratory diagnosis, informs the AFSVSPP (Administration of the Republic of Slovenia for Food Safety, Veterinary Sector and Plant Protection) regional office, and gives the owner additional instructions how to prevent the spread of infection. The diagnosis is confirmed at NVI (National Veterinary Institute) by pathomorphological examination, bacteriological examination and real-time PCR. The official veterinarian conducts an epidemiological investigation and implements the following measures: (1) prohibition of the movement of animals or their products; (2) euthanasia of all animals that fail a negative diagnostic test; (3) prohibition of slaughtering or opening the carcass; (4) vaccination of all ruminants and equines; (5) destruction of carcasses of dead animals; (6) destruction and disinfection of animal feces; (7) cleaning and disinfection of all equipment that has been in contact with infectious material; (8) disinfection of the ground where animals have died; (9) pest control (insects and rodents) and other farm sanitization measures (Hodnik et al. 2022). Nowadays, such an approach is implemented by more countries to protect the “One health” aspect.

In Bosnia and Herzegovina, cases of anthrax in ruminants have occurred sporadically over the past 25 years. The cutaneous form of the disease was reported in September 2014; two men have been identified while slaughtering cows and had come into contact with an infected animal carcass (Arapović et al. 2015). The latest case was reported in 2017 (Kozytska et al. 2023). Similarly, in southeastern Romania, two confirmed cases of anthrax were reported in October 2011, in a small town with a population of 3,400. One was a fatal case of cutaneous and anthrax meningitis, while the other was a case of cutaneous anthrax. In both cases people had come into contact with a single cow infected with *Bacillus anthracis* through the consumption of its meat or participation in the slaughter (Popescu et al. 2011). In the first case, a 20-year-old woman was taken to the hospital on October 7th with fever (40 °C), chills, malaise, pustular lesions on both forearms (onset of symptoms on October 6th ), blood pressure of 65/40 mm Hg and respiratory arrest (symptoms occurred on October 7th ). Three days after hospitalization, the patient developed symptoms of meningitis. On the same day, the patient died. During the hospital stay, the patient was mechanically ventilated and received intravenous dopamine, penicillin and ciprofloxacin. The cause of death was cardiovascular and respiratory failure and septic shock due to disseminated *B. anthracis* infection, confirmed by the presence of the bacteria on the skin and in the blood-stained cerebrospinal fluid (CSF). On the same day, another person from the same village was admitted with a confirmed case of cutaneous anthrax. The disease began on October 3rd. The patient developed pustules on his left index finger. The patient received intravenous penicillin and ciprofloxacin. The response to treatment was satisfactory. The general condition improved markedly and the patient was discharged from the hospital. In the same year, in Serbia, three cases of the cutaneous form of anthrax were only reported in June, in the autonomous province of Vojvodina. All cases were the result of infection during the slaughter of an anthrax-sick heifer. In the animal’s place of origin, anthrax was also found in other animals (Đurić et al. 2012). The latest cases have been reported on 21.08.2023, out of 3 people considered suspicious, two were hospitalized at the Târgu Mureș Emergency County Clinical Hospital and one at the Mureș County Clinical Hospital (https://flutrackers.com/forum/).

In Turkey, as a transcontinental country located in Southeastern Europe and Southwestern Asia, a total of 6873 human cases of anthrax were reported between 1995 and 2005. The mortality rate was 0.38–0.77 per 100,000 population. In 2000, the number of human anthrax cases was found to be 396. Since then, it has been steadily declining. For example later, in 2010, only 93 cases were noticed in the agricultural areas of eastern Anatolia. The disease was on average three times more frequent there than in the central part of that province. Another study by Turkish authors found that the cutaneous form of anthrax was present in 96.9% of people, while gastrointestinal and meningitis in resp. 1.9% and 1.2% (Doganay and Metan 2009). Penicillin was found to be effective in anthrax treatment; 88.7% of patients receiving penicillin G have recovered. The mortality rate was low, representing 2.8%. It is worth mentioning that 95.2% of people had direct contact with infected biological material (Doganay and Metan 2009). More recently (2019), an acute cutaneous form of anthrax in a patient was described (Kuloğlu et al. 2019). In cattle, between 1998 and 2004, 1304 cases of anthrax were reported; 882 cases died. During the same period, 556 cases of the disease were reported in sheep, and 518 animals died (Durmaz et al. 2012).

In Greece, one case has been identified in August 2005, while four cases occurred in mid-summer 2011. According to Gaitanis et al. 2016, the appearance of unexpected clusters of anthrax cases during dry periods in Southern Europe underscores the risk of a future recurrence of the disease (Gaitanis et al. 2016). The latest 2 anthrax cases have been reported in 2017 (Kozytska et al. 2023).

A literature study covering the years 2001–2014 on clusters of anthrax cases shows that half of the cases of the disease occurred in the countries of the Balkan Peninsula. Moreover, analyzing historical data on the incidence of anthrax in humans worldwide between 1924 and 1953, it is noteworthy that the incidence of the disease increased significantly in countries such as Turkey, Romania, Bulgaria and Yugoslavia. For example, between 1951 and 1953, 914–1500 cases of anthrax were recorded by these countries (Glassman 1958).

In 2012, in Bulgaria, one case of a cutaneous form of anthrax has been reported, as a consequence of the slaughter of a sheep. As a result of the deterioration of the general condition, the patient has been spatialized after one day administration of the doxycycline; the first symptoms appeared 3 days before hospitalization. The patient (32 years old) went to the hospital with forearm pain, fever, profuse sweating, and malaise. Initially, the disease has manifested by the appearance of a “pimple” on the right forearm and, subsequently, as a result of scratching, has generated congestion which led to swelling of the skin and forearm. Moreover, the patient’s dog has eaten the entrails of the slaughtered sheep and soon died. Occupation – mechanic. (Petkova et al. 2014). Later, in 2017, the European Centre for Disease Prevention and Control published a new case of anthrax in Bulgaria and 5 cases in their neighbor, Romania.

In Russian Federation, from 2009 to 2018, the incidence of anthrax has been reported in 14 regions of 6 Federal Districts. Coming into contact with sick/dead animals or consumption of infected meat led to the appearance of human infection. Over this period, about twenty-three human anthrax outbreaks were identified; 3 fatalities have been registered. However, compared to the 1999–2008 period, a reduction of the cases has been noticed, about 1,6 times less. The latest anthrax case has been reported in 2022 (Kozytska et al. 2023). The Russian neighbouring countries have also been affected (e.g. Georgia, Kazakhstan, China, Kyrgyzstan and Ukraine). In all enumerated countries, outbreaks of infections have been reported among both humans and livestock (Ryazanova et al. 2019).

Based on the data published by the National Institute of Public Health, in Poland, no human cases of anthrax have been identified in the last 10 years (http://wwwold.pzh.gov.pl/oldpage/epimeld/index_p.html). In contrast, numerous cases of the disease, predominantly in its cutaneous form, were previously reported. In late 1990, an atypical cutaneous form of anthrax involving the skin of the face was detected in a pregnant woman (Zasada et al. 2014). The lesion was located in a potentially dangerous area, that is the upper part of the face. The site may be associated with respiratory or central nervous system complications. The patient recovered without complications after antibiotic therapy and local surgery. The fetus and subsequent labor and delivery were unaffected.

Moreover, several countries from Asia and Africa have felt the epizootiological and epidemiological instability regarding anthrax; a higher incidence of anthrax is reported in tropical countries, primarily in Asia, followed by Africa (Fig. 2) (Sahoo 2020). The disease is endemic in developing countries whose economy is mainly agriculture and livestock-dependent. Among these countries is also Pakistan. Ali and Ejaz in 2023 alert the need for a vaccination program once anthrax is considered one of the animal diseases of Pakistan’s priority. Moreover, the results of the National Epidemiological Survey of important livestock diseases showed that anthrax is among the leading cause of death among cattle, goats, and sheep in the desert and hilly areas (Aftab 2023; Ali and Ejaz 2023).

Anthrax wreaks disease amongst mammalian species worldwide and has an endemic distribution not only in Asia but also in Africa (Badri et al. 2022). The authors reported the anthrax attack rates around 15% in 2014 and 29% in 2017, with case fatality rates up to 5%. The research underlines the importance and risk of the anthrax contamination, sugesting the need for the implementation of the vaccination programs in the fight against anthrax at the same level as for COVID-19. As long as the epidemiological pattern of anthrax in Africa involves humans, livestock, wildlife, and the environment, *Bacillus anthracis* remains a disease of public health concern that serves to fuel the devastating effects of SARS-CoV-2 in African communities (Badri et al. 2022).

## Anthrax – epidemiology and forgotten zoonosis

Before characterizing the threats above, it is worth recalling the most essential knowledge about *Bacillus anthracis* and the epidemiology of anthrax as a zoonosis. The properties of *Bacillus anthrax* make this bacterium particular.

**Fig. 3:**
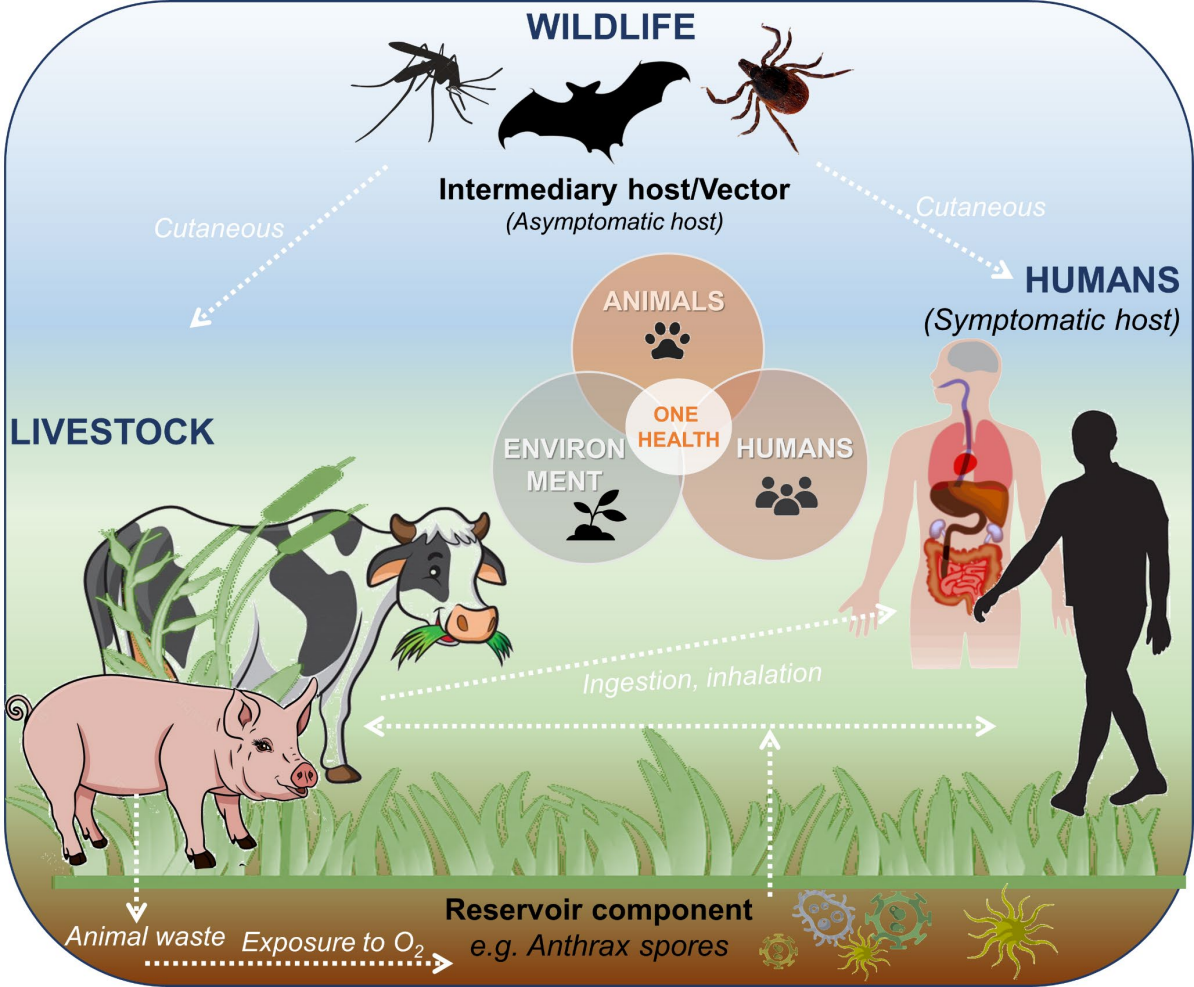
The transmission cycle of *Bacillus anthracis*

Figure 3 illustrates the possible transmission pathways of *Bacillus anthracis* in humans or mammals through ingestion, inhalation, or cutaneous pathways.

The common point of these complex paths are spores that originate from vegetation in excreted waste from cattle and can live in the environment for a long time (from several years to decades) and become activated when exposed to oxygen. Survival of spores in the soil is favored by the presence of organic compounds. Once they become active, they generate a series of severe illnesses. From another side, the transmission can result from direct contact (e.g. blood) with the infected insects (wildlife species) or indirectly through ingestion of the infected meat or fruit etc. The ingestion and inhalation paths are mostly fatal because, more often, it is difficult to recognize at the last stage, thus, it is laborious to administrate an effective treatment. In turn, in the case of untreated cutaneous cases, only 1% are fatal (Beyer and Turnbull 2009).

Herbivores are most susceptible to primary infections. Among livestock, the most vulnerable are ruminants (cattle, sheep, goats) as well as horses. Secondary infections in carnivores are associated with ingesting body parts from herbivores that have died of anthrax. As mentioned above, biting insects can transmit anthrax to animals. In herbivores, the disease progresses rapidly and often ends in death amid septicemia symptoms (Finke et al. 2020).

The milestone aspect of anthrax zoonosis approach is the way people are infected. Farmers, shepherds, butchers, sales representatives, veterinarians and workers handling and processing animal materials (hides, wool, meat, bones, etc.) most often suffer from anthrax. Infection by the droplet route is infrequent, occurring most frequently in perceivers of sheep. Additionally, cases of infection through coming into contact with clothing and objects made from the fur of infected and diseased animals have also been reported, and it is most likely that infection can occur during the intravenous use of psychoactive drugs (Finke et al. 2020).

Currently, besides West Africa, the Middle East, Central Asia (this area is called the anthrax belt) and Europe, anthrax cases, particularly as endemic infections, have also been observed in Mexico, Central and South America as well as the Balkans. In Central and Western Europe and the United States, anthrax cases in humans and animals are reported sporadically.

The number of human cases of anthrax in Poland since the beginning of the current century is only 7. The last three cases were confirmed in 2009. According to the National Institute of Public Health (http://wwwold.pzh.gov.pl/oldpage/epimeld/index_p.html), animal incidence has beenrare in recent years. The last case of the disease in an animal (a horse) was confirmed in 2014. The indicated number of cases of the disease is small, thus giving the impression that the threat from this disease to public health is practically non-existent.

It is essential to underline that it has also been supposed that not only has this threat not decreased, but, due to the changes occurring in the world in recent years, it will increase in Poland in the coming years. It refers to two potentially most dangerous aspects of these threats. The first is *Bacillus anthracis* as a biological weapon (bioterrorism) and the second is climate change, which also covers the area of our country.

## *Bacillus anthracis* as a biological weapon (bioterrorism)

Biological pathogens have long been considered as weapons of mass destruction. Many research centers around the world are investigating the use of microorganisms to cause disease in humans, animals, and plants to inflict damage on the enemy (Klietmann and Ruoff 2001). Although it is now possible to use genetic engineering achievements to modify or even create microorganisms for military purposes, *Bacillus anthracis* remains of interest as a potential biological weapon. This is due to the properties of this bacterium, the ease with which it can be produced in large quantities, and its many possible applications. These properties mean that laboratory conditions are not necessary for producing both vegetative forms and spores. Moreover, the spores of this microorganism in particular can be secretly transported around the world. Although, at present, the use of *Bacillus anthracis* as a biological weapon on a large scale may be considered highly unlikely, the use of this bacterium in a bioterrorist attack is not only possible, but has already taken place. In addition, these attacks have been effective, as they have led to the deaths not only of those targeted by these unlawful acts but also of bystanders (Gierczynski et al. 2004).

At the beginning of the current century, anthrax came into the consciousness of people worldwide due to sensational media reports of human cases of the disease in the United States (Sternbach 2003). It has been shown that the infections occurred using *Bacillus anthracis* as biological weapons (acts of bioterrorism). Bioterrorists sent the anthrax spore in properly crafted packages (mailing envelopes) through regular mail. Many people who came into contact with anthrax spores died, since, in the initial phase of the bioterrorist attack, anthrax was not considered a cause of the disease. Following the same route, attempts were also made in Poland; letters containing suspicious substance in the form of dust and powder were sent to various establishments. Indeed, no cases of biological pathogens were found in the shipments. However, modus operandi of the perpetrator indicated that these individuals knew how to interest or frighten state services responsible for the public health security and how to prevent the work of these services in combating potential attacks (Pardhoothman 2015). Modus operandi of the perpetrator of the suspected attacks indicated that this was not a series of random events, but actions aimed to test the efficiency of the state board against bioterrorist attacks. It is worth noting that these attacks took place after the previously mentioned successful attacks in the United States, as well as after the attack on the WTC towers in New York. The experience gained during the crackdown on bioterrorist attacks with the suspected use of carbide lasers made it possible to develop new procedures and improve the previous ones for readiness to combat such incidents and counter the potential consequences of such incidents.

On the other hand, the experience gained during counter-terrorism operations indicates that medical and veterinary professionals who do not have to deal with cases of this disease forget about the symptoms present in humans and animals. Therefore, infections or illnesses can lead to erroneous initial diagnoses, thus failing to notice the onset of an epidemic or bioterrorist attack. At this point, it should be strongly recalled that in the case of such a disease as anthrax, the moment of recognition of the disease (both in humans and animals) is crucial for effective control of the disease and prevention of its effects. Observations in recent years indicate that the appearance of numerous cases of the disease, which previously did not occur in a given area, not only causes difficulties in its control but also effectively disorganizes the efficiency of the state responsible for protecting public health (e.g. COVID-19 and ASF).

## *Bacillus anthracis* as a dormant threat

Leaving aside the unlawful activities involving *Bacillus anthracis*, it should be noted that there are increasing signs that the disease is returning to areas where it has not posed a threat for decades. It should be recalled that anthrax spores can survive in the soil for decades and, under favorable conditions, first infect herbivores, then other animals, and finally humans. This is most likely related to climate change (MAKSIMOVIC et al. 2017). Numerous anthrax cases in Siberia, where the permafrost is thawing, and recent cases in the Balkans confirm this regularity (Zasada et al. 2014).

In the same train of thought, the case reported in Croatia took place in July - the hottest month (38 °C, humidity exceeds 50%, pH about 5.8) in that area (Zasada et al. 2014). Therefore, the park creates the right conditions for anthrax spores, and, consequently, the development of the disease. Within two weeks, 107 cows died in the park; it is not to exclude the fact that the scale of infection and mortality among cattle will be more significant.

In the summer of 2016, Siberia experienced unprecedented heat. In July, an anthrax epizootic was found there (Yamal Peninsula, in about 1,500 reindeer) (Ezhova et al. 2021). The people to whom the animals belonged also became ill. About 40 people, including several children, were hospitalized. The illnesses are believed to have occurred due to thawing permafrost, including the carcass of an animal that died of anthrax during the 1941 epizootic. This chain of infections demonstrates emphatically the ability of anthrax spores to survive and retain the virulence of vegetative forms arising from these spores.

The Croatian and Siberian examples indicate the longevity of anthrax bacillus spores. Thus, they provide a kind of warning about the possibility of the appearance of this microorganism in places where it has not occurred for years. Moreover, considering modern economic ties and developing tourism (travel to remote areas of the world), the transfer of this biological pathogen to our country (Poland) should also be considered.

Climate change is causing glaciers to melt and land to thaw in far north areas. In addition, many wetlands and swampy areas are being drained. We do not know the epidemiological status of these areas in the past, and potentially any of them could be contaminated with anthrax spores (MAKSIMOVIC et al. 2017). These areas, like Lonjsko polje, are becoming pastures for animals, including livestock. This situation creates a potential danger of anthrax infections in humans and animals, even with an epidemic course. What is more, failure to recognize the threat could allow the movement of raw materials from and to the sick and infected animals to remote areas of the world, including Poland.

## Concluding remarks

The examples of real and potential risks of anthrax in Poland cited above entitle us to conclude that these infections can now be expected anytime and anywhere. In addition, infections can occur through previously unknown routes and in people not belonging to the existing risk groups. Examples are the cases of anthrax found in Scotland in drug addicts taking psychotropic drugs by the intravenous route or in people crafting drums from imported hides.

Because of the growing threats, institutions responsible for the health safety in our country should intensify training activities for medical and veterinary professionals. These trainings should also, and perhaps most importantly, include family doctors and free-practice veterinarians. It should be noted that state sanitary inspectors, except for the ones at the central level, are mostly non-doctors (including veterinarians), who may fail to perform proper epidemiological analyses.

Climate change is a fact. Progressive warming is observed in the northern hemisphere in particular. Thus, the epidemiological threat from anthrax will grow, including in Poland. The health security of our country’s population depends on the efficiency of institutions responsible for public health, and they must quickly and efficiently identify and combat threats from infectious diseases. In the case of zoonosis, this includes efficient interaction with the Veterinary Inspection authorities. It should not be forgotten that epidemiological and epizootic threats significantly affect the country’s economic development. The Covid-19 and ASF epidemics best illustrate these threats.

Anthrax is a dormant threat, the question is not if, but when it will threaten the health and lives of people and animals in Poland.

## Data Availability

The datasets generated during and/or analysed during the current study are available from the corresponding author on reasonable request.

## References

[CR3] Aftab J (2023) Anthrax (Bacterial Disease). Pak Dairy Info (Online Dairy Farming Guide). Accessed: April 2023. URL: https://www.pakdairyinfo.com/anthrax.htm

[CR1] Ali S, Ejaz M (2023). Anthrax in Pakistan. Ger J Microbiol.

[CR2] Arapović J, Skočibušić S, Jelavić B et al (2015) Two cases of human cutaneous anthrax in Bosnia and Herzegovina, September 2014. Eurosurveillance 20 10.2807/1560-7917.ES2015.20.7.21039 10.2807/1560-7917.es2015.20.7.21039 25719961

[CR4] Badri R, Uwishema O, Wellington J (2022). Anthrax outbreak amidst the COVID-19 pandemic in Africa: challenges and possible solutions. Annals of Medicine and Surgery.

[CR5] Beyer W, Turnbull PCB (2009). Anthrax in animals. Mol Aspects Med.

[CR6] Blevins SM, Bronze MS (2010). Robert Koch and the ‘golden age’ of bacteriology. Int J Infect Dis.

[CR7] Cepas V, Soto SM (2020). Relationship between virulence and resistance among Gram-negative Bacteria. Antibiotics.

[CR8] Derzelle S, Thierry S (2013). Genetic diversity of Bacillus anthracis in Europe: genotyping methods in forensic and epidemiologic investigations. Biosecur Bioterror.

[CR9] Doganay M, Metan G (2009). Human Anthrax in Turkey from 1990 to 2007. Vector-Borne and Zoonotic Diseases.

[CR10] Đurić P, Ćosić G, Rajčević S et al (2012) Three probable cases of cutaneous anthrax in autonomous province of Vojvodina, Serbia. J Euro 17. 10.2807/ese.17.01.20050-en 22264812

[CR11] Durmaz R, Doganay M, Sahin M (2012). Molecular epidemiology of the Bacillus anthracis isolates collected throughout Turkey from 1983 to 2011. Eur J Clin Microbiol Infect Dis.

[CR12] Ehling-Schulz M, Lereclus D, Koehler TM (2019) The Bacillus cereus Group: Bacillus Species with Pathogenic Potential. Microbiol Spectr. May;7(3):10.1128/microbiolspec.GPP3-0032-2018. 10.1128/microbiolspec.gpp3-0032-2018 PMC6530592 31111815

[CR13] Ezhova E, Orlov D, Suhonen E (2021). Climatic factors influencing the Anthrax outbreak of 2016 in Siberia, Russia. EcoHealth.

[CR14] Finke E-J, Beyer W, Loderstädt U, Frickmann H (2020). Review: the risk of contracting anthrax from spore-contaminated soil – a military medical perspective. Eur J Microbiol Immunol (Bp).

[CR15] Gaitanis G, Lolis CJ, Tsartsarakis A (2016). An aggregate of four Anthrax cases during the dry summer of 2011 in Epirus, Greece. Dermatology.

[CR16] Gierczynski R, Kaluzewski S, Rakin A (2004). Intriguing diversity of Bacillus anthracis in eastern Poland the molecular echoes of the past outbreaks. FEMS Microbiol Lett.

[CR17] Glassman HN (1958). World incidence of Anthrax in Man. Public Health Rep.

[CR18] Goel AK (2015). Anthrax: a Disease of biowarfare and public health importance. World J Clin Cases.

[CR19] Habrun B, Racic I, Kompes G (2011). The antimicrobial susceptibility and virulence factors of Bacillus anthracis strains isolated in Croatia. Vet Med (Praha).

[CR20] Hodnik JJ, Knific T, Starič J et al (2022) Corrigendum: overview of Slovenian control programmes for selected cattle Diseases, listed under category C, D or E of the European Animal Health Law. Front Vet Sci 8. 10.3389/fvets.2021.835395 10.3389/fvets.2021.835395 PMC8790675 35097058

[CR21] Klietmann WF, Ruoff KL (2001). Bioterrorism: implications for the clinical microbiologist. Clin Microbiol Rev.

[CR22] Koehler TM (2009). Bacillus anthracis physiology and genetics. Mol Aspects Med.

[CR23] Kozytska T, Bassiouny M, Chechet O (2023). Retrospective analysis of Official Data on Anthrax in Europe with a special reference to Ukraine. Microorganisms.

[CR24] Kuloğlu F, Gözübüyük AA, Kara M, Akata F (2019). Cutaneous Anthrax Outbreak in the Trakya Region of Turkey. Balkan Med J.

[CR26] Maksimovic Z, Cornwell MS, Semren O, Rifatbegovic M (2017). The apparent role of climate change in a recent anthrax outbreak in cattle. Revue Scientifique et Technique de l’OIE.

[CR25] Manzulli V, Fasanella A, Parisi A (2017). Evaluation of in vitro antimicrobial susceptibility of Bacillus anthracis strains isolated during anthrax outbreaks in Italy from 1984 to 2017. J Vet Sci.

[CR27] Moayeri M, Leppla SH, Vrentas C (2015). Anthrax Pathogenesis. Annu Rev Microbiol.

[CR28] Pardhoothman S (2015) An analysis of the modus operandi of perpetrators in human trafficking, University of South Africa, Pretoria. http://hdl.handle.net/10500/21167

[CR29] Petkova T, Popivanov I, Doichinova T (2014). Cutaneous Anthrax - Contemporary Clinical and Epidemiological aspects. Balkan Military Medical Review.

[CR30] Popescu R, Pistol A, Miltaru L et al (2011) Two cases of Infection with Bacillus anthracis, Romania, October 2011. Eurosurveillance 16. 10.2807/ese.16.45.20008-en 22114977

[CR31] Ryazanova AG, Ezhlova EB, Pakskina ND et al (2019) Epidemiological Situation on Anthrax in 2018, the Forecast for 2019. Probl Particularly Danger Infections 98–102. 10.21055/0370-1069-2019-1-98-102

[CR32] Sahoo KC, Negi S, Barla D et al (2020) The landscape of anthrax prevention and control: stakeholders’ perceptive in Odisha, India. Int J Environ Res Public Health 17. 10.3390/ijerph17093094 10.3390/ijerph17093094 PMC7246808 32365539

[CR33] Schmid G, Kaufmann A (2002). Anthrax in Europe: its epidemiology, clinical characteristics, and role in bioterrorism. Clin Microbiol Infect.

[CR34] Sternbach G (2003). The history of anthrax. J Emerg Med.

[CR35] Sushma B, Shedole S, Suresh KP et al (2021) An Estimate of Global Anthrax Prevalence in Livestock: a Meta-analysis. Vet World 1263–1271. 10.14202/vetworld.2021.1263-1271 10.14202/vetworld.2021.1263-1271 PMC8243666 34220129

[CR36] Weekes C, Kotra LP (2007) Bacillus Infections. xPharm: the Comprehensive Pharmacology Reference. Elsevier, pp 1–7

[CR37] Zasada AA, Formińska K, Ogrodnik A (2014). Screening for anthrax occurrence in soil of flooded rural areas in Poland after rainfalls in spring 2010. Ann Agric Environ Med.

